# Pair-Rule Gene Orthologues Have Unexpected Maternal Roles in the Honeybee (*Apis mellifera*)

**DOI:** 10.1371/journal.pone.0046490

**Published:** 2012-09-28

**Authors:** Megan J. Wilson, Peter K. Dearden

**Affiliations:** Laboratory for Evolution and Development, National Research Centre for Growth and Development and Genetics Otago, Biochemistry Department, University of Otago, Dunedin, New Zealand-Aotearoa; University of Iceland, Iceland

## Abstract

Pair-rule genes are a class of segmentation genes first identified in *Drosophila melanogaster*. In *Drosophila*, these genes act to translate non-periodic information produced by the overlapping patterns of gap gene expression into patterns of gene expression in every other segment. While pair-rule genes are, for the most part, conserved in metazoans, their function in pair-rule patterning is not. Many of these genes do, however, regulate segmentation in arthropods and do so with dual-segment periodicity. Here we examine the expression and function of honeybee orthologues of *Drosophila* pair-rule genes. Knockdown of the expression of these genes leads to extensive patterning defects, implying that they act in early patterning, as well as segmentation in honeybee embryos. We show that these pair-rule gene orthologues indeed regulate the expression of honeybee maternal and gap genes implying roles in maternal patterning of the honeybee embryo.

## Introduction

Pair-rule genes are a set of genes identified in *Drosophila melanogaster* that act in segmentation [1]. Pair-rule genes translate non-segmental information, from the overlapping gradient expression domains of gap and maternal coordinate genes, into segmental information (Reviewed in [2]). The overlapping periodic expression domains of pair-rule genes lead to the segmental expression of segment polarity genes, which act to initiate and maintain the parasegment boundary, effectively completing segmentation. Mutations in pair-rule genes cause different defects in adjacent segments, for example *even-skipped* (*eve*) causes loss of each even-numbered segment [1]. Pair-rule genes are often expressed in a pattern consistent with their function, *eve*, for example, is expressed in even-numbered segments [3].

In other arthropods the expression patterns and function of pair-rule genes have been difficult to interpret [4]–[15]. The orthologs of *Drosophila* pair-rule genes are often not expressed in a classical pair-rule pattern, either not being expressed in any pattern similar to pair-rule [4], [9], or being expressed with ‘dual segment periodicity’ [12], [15]. In these latter cases the genes are often expressed in broad domains covering two segments, with the expression patterns splitting to form segmental stripes. The function of these genes in non-drosophilid insects has often been difficult to determine due to the lack of tools available to manipulate gene expression.

The most extensive analysis of pair-rule gene orthologue function outside *Drosophila* has been carried out in the beetle *Tribolium castaneum* [11], [16]. In this species the orthologues of the primary pair-rule genes *eve*, *runt* (*run*) and *odd-skipped* (*odd*) act as pair-rule genes, but produce asegmental embryos when knocked down because they activate each other's expression. They also show that orthologues of *paired* (*prd*) and *sloppy-paired* (*slp*) act as secondary pair-rule genes whereas *hairy* (*h*), *fushi-taratzu* (*ftz*), *odd-paired* and *Tenacin-major* do not act as pair-rule genes at all.

Expression patterns suggesting pair-rule or dual segment periodicity patterning, especially in orthologues of *Drosophila* pair-rule genes, have also been found in *Schistocerca gregaria* locusts [12], the cricket *Gryllus bimaculatus* [7], the spider mite *Tetranychus urticae* [14] and in the myriapod *Strigamia maritima* [17]. This is not to say that these genes are expressed in these patterns, or have pair-rule functions, in all arthropods. In locusts *eve* and *ftz* are expressed in a posterior domain [4], [9], in the milk-weed bug *Oncopeltus fasciatus eve* has no pair-rule function, but does act as a gap gene [6]. In the crustacean *Sacculina carcini, ftz* is expressed only in the nervous system [18], and in the myriapod *Lithobious forficatus*, *eve* is expressed in a posterior domain and a few segmental stripes of cells during segmentation [19].

It seems that while pair-rule patterning is conserved in arthropods, many orthologues of *Drosophila* pair-rule genes often do not act in segmentation, or have other roles in development.

Here we describe the expression patterns and functions of four honeybee pair-rule gene orthologues, *fushi-taratzu*, *even-skipped*, *runt* and *hairy*. In honeybee three pair-rule orthologous gene expression patterns have been previously described, *prd* [15], *eve* [20], [21] and *ftz* [22] but no functional analysis has taken place. Honeybees develop in a long-germ band mode, similar to *Drosophila* [23]. The currently accepted phylogeny of holometabolous insects, however, implies that long germ development in these two species evolved independently [24], [25]. This gives us the opportunity to examine the function of pair-rule genes in a distantly related, but morphologically similar, embryo.

Here we report that each of these genes is expressed with dual segment periodicity during honeybee segmentation but that, remarkably, three of these genes are also expressed maternally and have functions that affect the expression of maternal coordinate and gap genes in honeybees.

## Materials and Methods

### Cloning of *eve*, *hairy*, *ftz* and *run from A. mellifera*

The cloning of *Am-eve*, *Am-ftz*, *Am-run*, *Am-prd*, *Am-gt*, *Am-cad*, *Am-kr*, *Am-otd-1* and *Am-hb* has been reported previously [20], [22], [26], [27], A fragment of *Am-h* coding sequence was amplified using the following oligonucleotide primers TCCCCCGCGCCGACCTC, *Amh*5′ & TTTCTCCTCCACCTCCCGCACCAC, *Amh*3′. Details of all genes discussed in this work can be found in Table S1.

### Whole-mount *in situ* hybridization to honeybee embryos and queen ovaries

*In situ* hybridization on honeybee embryos or queen ovarioles was carried out as described in [28]. Embryos were counterstained with DAPI and mounted in 70% glycerol. Images were captured on an Olympus BX61 microscope with a DP71 camera. Embryos were staged as per [29]. In all cases control embryos and ovaries, stained with sense probes for each gene, were examined and found to have no specific staining.

### RNAi-mediated knockdown in honeybee embryos

dsRNA was produced from cDNAs of *eve*, *h*, *run* and *ftz* cloned into pLitmus 38i (NEB) using the MEGAscript RNA kit (Ambion). RNAi was performed as described in [30], [31]. Embryos were injected at 1–4 hours after egg laying (before cellularisation). Injected embryos were incubated at 35 C and 80% humidity for 24 (stage 4), 30 (stage 5), 48 hours (stage 9) or to hatching (70 hours later). For each target, 100–400 embryos were injected.

## Results

### Expression and RNAi phenotypes of honeybee pair-rule gene orthologues

**Figure 1:**
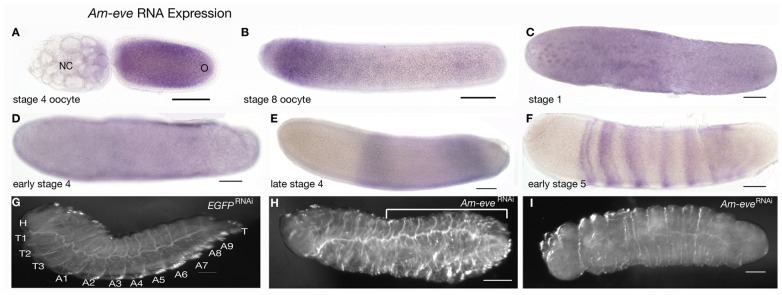
Expression and function of *Am-eve*. Embryos are oriented with anterior left and dorsal up. Scale bars are 200 µm. (A) Expression of *Am-eve* in a stage 4 oocyte, mRNA is present in the oocyte (O) and in posterior nurse cells (NC). (B). In stage 8 oocytes *Am-eve* mRNA is enriched in the anterior pole. (C) In newly laid embryo, *Am-eve* RNA is present throughout the embryo but enriched around energids. (D) Early stage 4 embryo with ubiquitous *Am-eve* RNA. (E) Late stage 4 embryo, *Am-eve* RNA is present in a broad abdominal domain and absent from both anterior and posterior. (F) Stage 5 embryo, just prior to gastrulation, *Am-eve* is expressed in distinct broad stripes of cells along the anterior –posterior axis, with anterior stripes beginning to split. (G). A just hatched larva injected with EGFP dsRNA with segments labeled. (H) and (I) *Am-eve* ^RNAi^ larva showing fusion of central, posterior and terminal segments (bar in H) or asegmental larvae (I).

**Figure 2:**
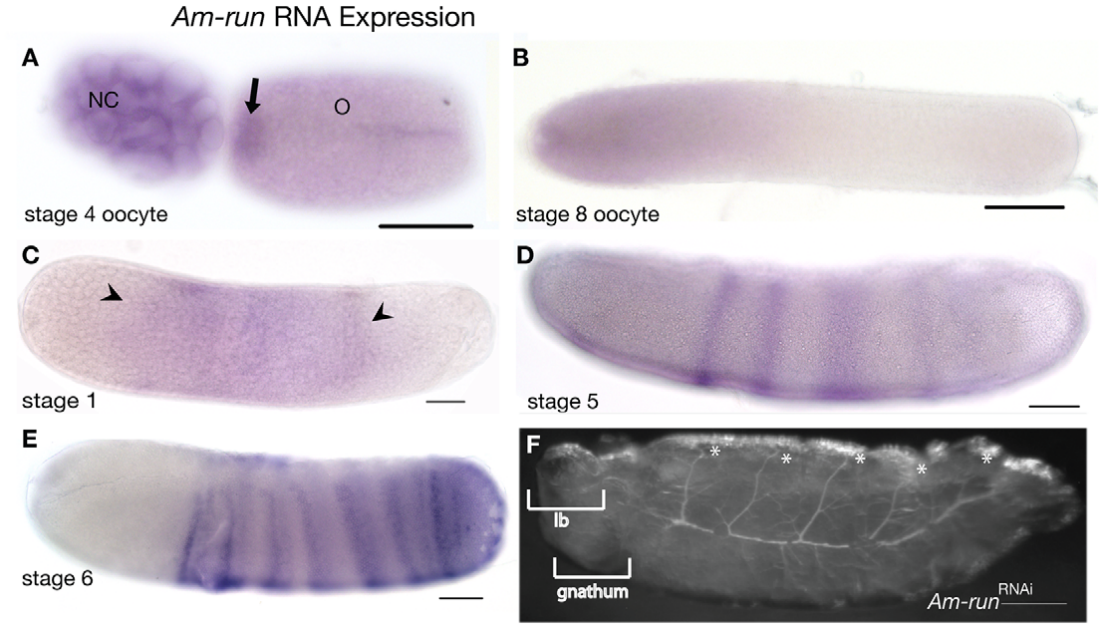
Expression and function of *Am-run*. Embryos are oriented with anterior left and dorsal up. Scale bars are 200 µm. (A) Expression of *Am-run* in a stage 4 oocyte, dorsal view. *Am-run* RNA is present in both nurse cells (NC) and the oocyte (O), where it is enriched at the anterior of the oocyte (arrow), and in a stripe along the oocyte surface. (B) *Am-run* RNA is enriched in the anterior of late stage oocytes. (C) In stage 2 embryos, *Am-run* RNA is present in a faint broad domain in central regions of the embryos, marked by arrowheads in C. (D) Mid stage 4 embryo with stripes of cells expressing *Am-run* RNA appearing across the anterior–posterior axis. (E) By gastrulation (stage 6) distinct stripes of cell expressing *Am-run* mRNA are detected throughout thoracic and abdominal regions. These stripes of cells often display darker staining in cells at the anterior of each stripe and then split as stage 6 progresses. (F) *Am-run* ^RNAi^ larva, showing disrupted thoracic and abdominal segments. Segments appear more widely spaced than in controls, as implied by the pattern of trachea, perhaps indicating loss of alternate segments. Larvae also show expansion of the labrum (lb) and loss of head appendages (gnathum). Asterisks mark segmental trachea.

**Figure 3:**
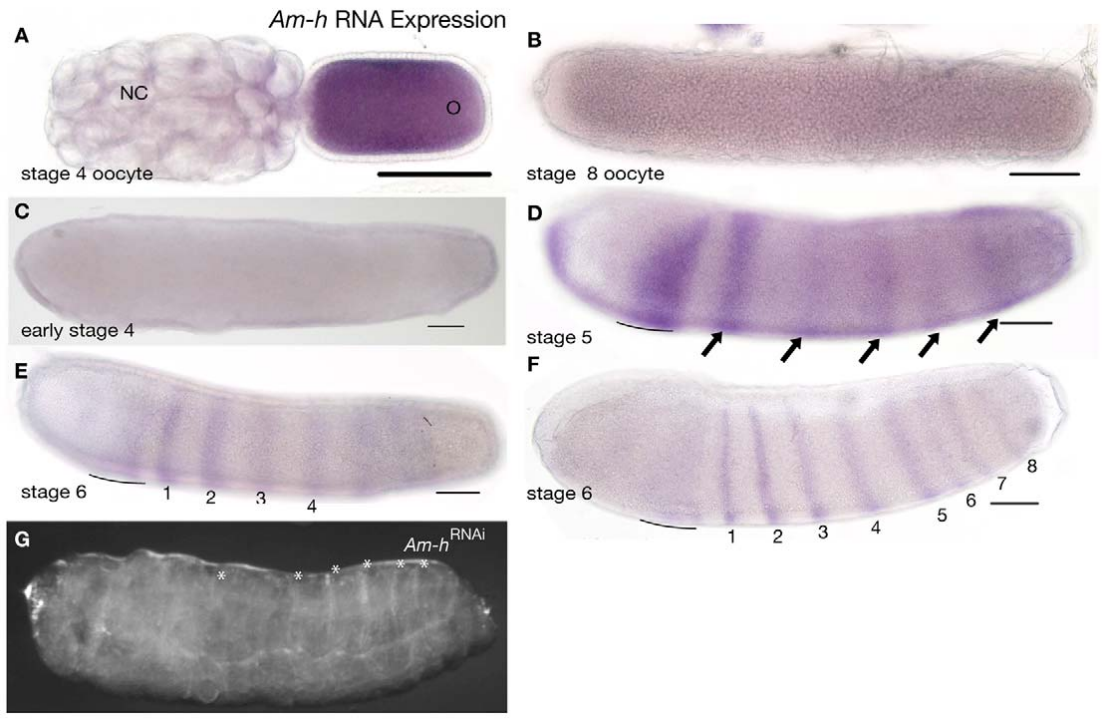
Expression and function of *Am-h*. Embryos are oriented with anterior left and dorsal up. Scale bars are 200 µm. (A) *Am-h* RNA expression in a stage 4 oocyte (O) and, more weakly, in nurse cells (NC). (B) Mature oocyte showing faint staining for *Am-h* RNA. (C) Stage 4 embryo showing no staining for *Am-H* RNA. (D) Zygotic *Am-h* RNA appears in a broad anterior-thoracic domain (line in D, E and F) and stripes of cells expressing *Am-h* begin to appear, in anterior-posterior order, during stage 5 (D) and 6 (E and F). By late stage 6 (gastrulation, (F)), eight stripes of *Am-h* RNA expression, and a posterior cap of cells expressing *Am-h* RNA, is detected. (G) *Am-h* ^RNAi^ larvae showing loss and disruption of abdominal segments. Asterisks mark segmental trachea.

**Figure 4:**
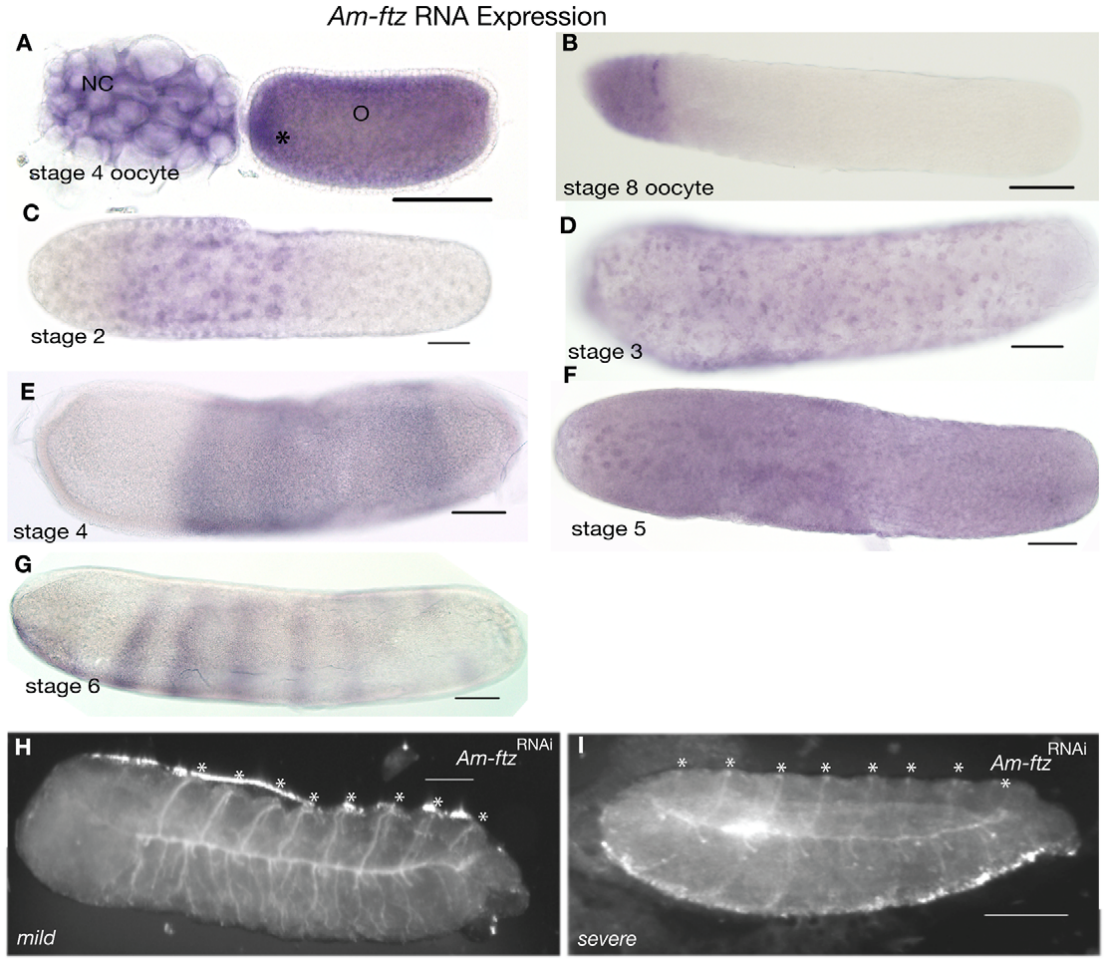
Expression and function of *Am-ftz*. Embryos are oriented with anterior left and dorsal up. Scale bars are 200 µm. (A) *Am-ftz* RNA expression in a stage 4 oocyte; RNA is present in both oocytes (O) and nurse cells (NC). RNA in the oocyte is concentrated towards the anterior (asterisk in A). (B) Late stage 8 oocyte with *Am-ftz* RNA present only in the anterior regions. (C) Early embryo, stage 2, *Am-ftz* RNA is associated with nuclei as they appear at the surface of the egg, spreading down the embryo as development proceeds (D). (E) Stage 4 embryo displaying *Am-ftz* RNA in a broad central abdominal domain, with RNA absent form both anterior and posterior. (F) In stage 5, the abdominal domain divides into stripes of cells expressing *Am-ftz* RNA such that, by stage 6, seven clear stripes of cells expressing *Am-ftz* RNA are present (G). (H–I). *Am-ftz* ^RNAi^ larvae have defects in pattern from the anterior, ranging from loss of the head (H), to loss of all but posterior abdominal segments (I). Asterisks mark segmental trachea.

To determine the domains of expression and function of honeybee pair-rule gene orthologues we examined the RNA expression and RNA interference knockdown phenotypes of *Am-eve*, *Am-runt*, *Am-h* and *Am-ftz* (Figures 1, 2, 3, 4).

*Am-eve* RNA is present in the ovarioles of the queen ovary, in maturing oocytes and in the posterior nurse cells (Figure 1A). In about-to-be laid oocytes, *Am-eve* RNA becomes enriched at the anterior pole (Figure 1B). In early embryos (stage 1–4) *Am-eve* mRNA is distributed through the embryo, and enriched around energids at early stages (Figure 1C) and generally throughout the embryo as it cellularises (Figure 1D). As stage 4 progresses *Am-eve* RNA is lost from both anterior and posterior poles of the embryo leaving a broad domain of expression, modulated with stronger expression at the anterior and posterior ends of the domain, in central regions of the embryo (Figure 1E). In late stage 4 and the beginning of stage 5, broad stripes of cells begin to express *Am-eve* RNA in trunk regions of the embryo and then split, with new broad stripes appearing in anterior-posterior sequence, and then splitting as the next broad stripe appears (Figure 1F and [20]).

To determine the role of *Am-eve* in honeybee embryogenesis we injected double stranded RNA (dsRNA) targeting *Am-eve* into just-laid embryos and then incubated these embryos until hatching (72 hours later). Compared to control RNAi injections (Figure 1G), *Am-eve* ^RNAi^ produces phenotypes ranging from individuals with fused posterior segments and an absent terminal segment (marked by bar in Figure 1H) to asegmental larvae with a distinct head with mouthparts as the only identifiable morphology (Figure 1I).

*Am-run* RNA is detected in the ovary in mid-stages, where RNA is expressed by the nurse cells and present in an anterior domain, and a stripe along one side of the oocyte (Figure 2A). In just-about-to-be-laid oocytes (Figure 2B) *Am-run* RNA is present only in anterior regions. In early embryos, *Am-run* RNA is present in a very faint domain in abdominal regions (Figure 2C). By stage 5, broad stripes of cells begin to express *Am-run* RNA in anterior-posterior sequence along the embryo (Figure 2D), which then split as development proceeds (Figure 2E and [27]).

Knockdown of *Am-run* expression resulted in larvae with defective segmentation (Figure 2F). The majority of *Am-run* ^RNAi^ larvae have only slight indications of segmentation, particularly reflected in the organization of trachea. Segmentally-organised trachea are less densely spaced, perhaps indicating a loss of alternating segments, or an expansion of remaining segments. The labrum is enlarged (lb) and gnathal appendages are absent (Figure 2F).

*Am-hairy* mRNA is detected in the ovary in mid-stage oocytes, with faint RNA expression in the nurse cells and strong staining for RNA throughout the oocyte (Figure 3A). This RNA staining is greatly decreased in just-about-to-be-laid oocytes (Figure 3B) and is absent from early embryos up to stage 4 (Figure 3C). Zygotic expression of *Am-h* RNA is first detected late in stage 5 as a broad thoracic stripe, quickly joined by thinner stripes in anterior to posterior sequence (arrows in Figure 3D). During stage 6, eight stripes of *Am-hairy* form in anterior to posterior sequence in abdominal regions (Figure 3E–F).

*Am-h* ^RNAi^ injected embryos produce larvae with fused thoracic and anterior abdominal segments, with many larvae showing fusion of all segments (Figure 3G).

*Am-ftz* RNA is expressed maternally in oocytes and nurse cells at mid-stages of oogenesis (Figure 4A). *Am-ftz* mRNA comes to be enriched at the anterior pole of mature oocytes (Figure 4B). *Am-ftz* RNA is associated with energids as they populate the egg surface after laying, causing redistribution of the anterior maternal RNA (stage 2, Figure 4C and D). By late stage 4, *Am-ftz* RNA is expressed in a broad abdominal domain of cells and is absent from cells at the anterior and posterior poles (Figure 4E). Expression in this broad domain first becomes modulated and later splits (stage 5) to form seven broad stripes of cells expressing *Am-ftz* RNA (Figure 4F, G and [22]).

Injection of dsRNA targeting *Am-ftz* results in larvae with absent anterior segmentation and head patterning but with clear thoracic and abdominal segments (Figure 4H and 4I). These phenotypes are similar to those obtained with weak knockdown of the anterior-patterning genes, *Am-otd1* and *Am-hb* [32].

The larval phenotypes of honeybee RNAi knockdown experiments are often difficult to interpret because the cuticle is weak and has few landmarks that allow segments to be distinguished. To better interpret the phenotypes seen in our RNAi knockdown experiments we examined their effects on segmentation gene expression.

### Segment polarity gene expression in pair-rule gene knockdown embryos

**Figure 5:**
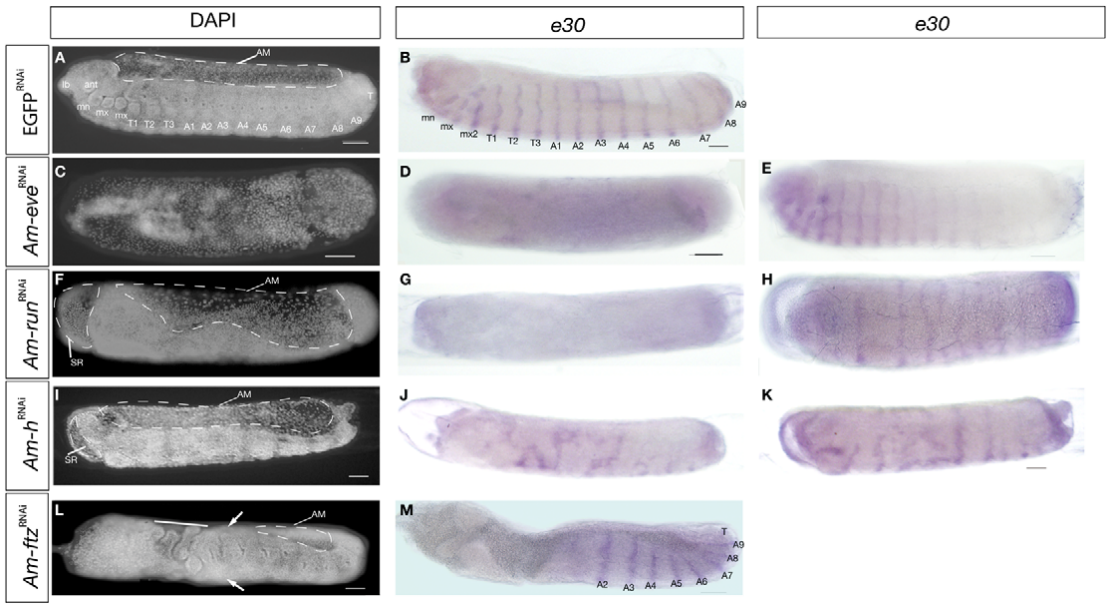
DAPI and engrailed (*e30*) staining RNAi injected stage 9 embryos. All embryos are oriented with anterior left and dorsal side up. Scale bars are 100 µm. (A) DAPI stained EGFP embryo, segments are labeled. B) EGFP^RNAi^ embryo stained for *e30* RNA, which marks the posterior of each segment. C and D). Severely affected *Am-eve* ^RNAi^ embryo stained with DAPI (C) or *e30* RNA (D) showing complete loss of segmentation. (E) More mild phenotype induced by *Am-eve* ^RNAi^. In this specimen, posterior stripes of e30 are reduced in intensity or absent. (F) DAPI stained or (G) *e30* RNA stained severly affected *Am-run* ^RNAi^ embryos have unsegmented germ bands and extension of the extra-embryonic membranes. (H) More mildly affected embryos show reduction in intensity and disorganization of *e30* stripes. (I) DAPI or (J) *e30* stained severely affected *Am-h^RNAi^* embryos showing expansion of extra-embryonic membranes and loss of both segmental morphology and *e30* RNA stripes. (K) More mildly affected embryos display disorganized e30 stripes. (L) DAPI stained or (M) *e30* RNA stained *Am-ftz* ^RNAi^ showing loss of anterior segmentation, disorganized central segments and reduction of the amnion. No more mildly affected individuals occurred with this injection. Abbreviations: serosa (SR), amnion (AM).

Pair-rule genes in *Drosophila* feed patterning information forward into the segment polarity network. By examining the expression of a marker segment polarity gene, *engrailed* (named *e30* in honeybee [33]), we aimed to determine if segment polarity gene expression is affected by pair-rule gene knockdown. We stained the nuclei of stage 9 embryos, at the end of the segmentation process, with DAPI and examined the expression of *e30* RNA, which marks the anterior compartment of each parasegment (Figure 5A and B).

In all our RNAi experiments, except those targeting *Am-ftz*, a range of phenotypes was produced. Figure 5 presents both severe examples of the phenotypes (D, G, J and M) as well as more mild effects (E,H and K).

In severely affected *Am-eve* ^RNAi^ stage 9 embryos (Figure 5C and D) no expression of *e30* appears (Figure 5D) and the embryos have considerably fewer cells as determined by DAPI staining (Figure 5C). More mild phenotypes have clear stripes of cells expressing *e30* RNA, with loss or weakened expression only in the stripes marking posterior segments (Figure 5E).

Severely affected *Am-run* ^RNAi^ embryos (Figure 5F and G) also have no expression of *e30* RNA (Figure 5F). In DAPI stained embryos the germ band is distinguishable but is not visibly segmented (Figure 5F). DAPI staining also reveals defects in the extra-embryonic membranes of *Am-run* ^RNAi^ embryos. In wild-type stage 9 embryos, the amnion is visible over the dorsal surface of the yolk (Figure 5A). In *Am-run* ^RNAi^ embryos, the amnion is expanded, particularly in the posterior (Figure 5F). The serosa, normally removed before staining, is located at the anterior (distinguishable from embryonic tissue due to its large, widely spaced nuclei), and fails to expand to envelope the embryo (Figure 5F). In more weakly affected embryos (Figure 5H), the serosa envelops the embryo, as in control embryos, and stripes of *e30* RNA expressing cells are visible in the germ-band, but are less ordered than in control injected embryos.

In severely *Am-h* ^RNAi^ embryos (Figure 5I and J) anterior stripes of *e30* RNA are absent, but disorganized expression is seen in central regions (Figure 5J) where the abdominal stripes would normally be present. Staining with DAPI confirms loss of anterior and abdominal segments (Figure 5I). As in *Am-run* ^RNAi^ treated embryos, the serosa has failed to expand in *Am-h* ^RNAi^ embryos, but the amnion is present and slightly expanded in the posterior (Figure 5I). In more mildly affected embryos, disorganised stripes of *e30* RNA are present throughout the germband (Figure 5K).

*Am-ftz* ^RNAi^ embryos stained with DAPI (Figure 5L) display loss of patterning in the anterior, irregular segments in thoracic regions, central abdominal segments now extend further towards the dorsal side of the embryo, while posterior segments appear normal. Staining for *e30* mRNA staining indicates that posterior segments, identified by their tracheal pits (Figure 5L) and spaced as in control embryos, are present in *Am-ftz* ^RNAi^ embryos (Figure 5M). No mild versions of this phenotype occurred in our *Am-ftz* ^RNAi^ experiments.

These experiments demonstrate that knockdown of these pair-rule gene orthologues have profound affects on *e30* RNA staining. *Am-h* RNAi embryos have a phenotype consistent with roles in segmentation for these genes. The phenotypes of the other pair-rule orthologues are also consistent with patterning roles earlier in development, as for *Am-ftz*, obscuring somewhat their later roles in segmentation, some of which may produce pair-rule-like phenotypes (*Am-run*, Figure 2F), potential pair-rule like modulation of *e30* RNA stripes (Figure 5H and K)), or more generally in segmentation as implied by the lack of *e30* RNA staining in *Am-eve* ^RNAi^ and *Am-run* ^RNAi^ embryos.

The defects in extra-embryonic membranes in these specimens imply that our RNAi experiments are disrupting the formation and/or patterning of these membranes. We thus examined the expression of a marker of extra-embryonic fate, *Am-zen* [22], [32], at stage 5 in control and injected embryos. Knockdown of any one of our honeybee pair-rule gene orthologues causes significant changes in *Am-zen* expression and morphology of the extra-embryonic membranes at early stages (Figure S1).

### Pair-rule gene expression in pair-rule orthologue knockdown embryos

**Figure 6:**
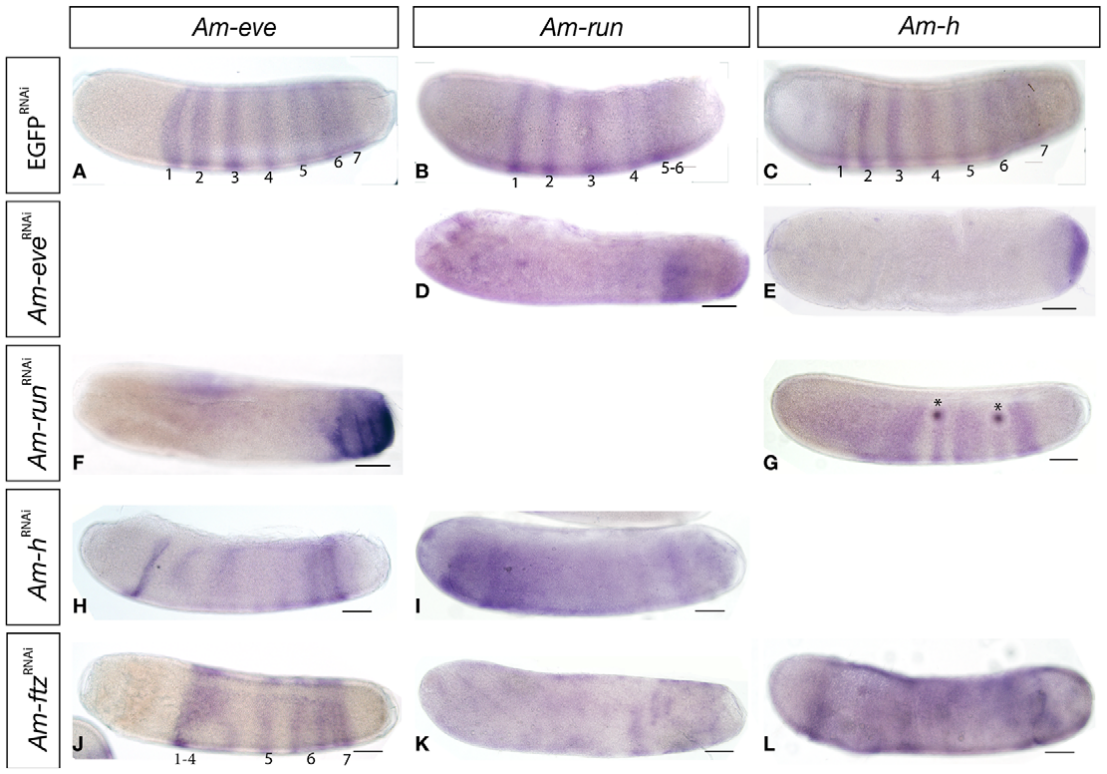
Impact of pair-rule gene orthologue knockdown on pair-rule expression. All embryos are oriented with anterior left and dorsal side up. Scale bars are 200 µm. Expression of *Am-eve* (A), *Am-run* (B) and *Am-h* (C) *Am-run* at stage 5–6 appears as six or seven broad stripes across the anterior-posterior axis of the embryo. These stripes subsequently split as development proceeds. *Am-ev*e^RNAi^ stage 5 embryo stained for *Am-run* (D) showing loss of all but an abdominal stripe, broader than in controls. *Am-h* (E) stained *Am-ev*e^RNAi^ stage 5 embryo showing loss of all segmental stripes and a posterior terminal cap of RNA expression. (F) *Am-eve* expression in an *Am-run^RNAi^* embryo showing expression only inn the post posterior abdominal stripes. (G). *Am-h* stripes are disorganized in *Am-run^RNAi^* embryos, forming a broad anterior domain, followed by two sets of a narrow stripe, then a broad one along the length of the embryo. Dark spots (asterisks) are damage to the other side of the embryo (H) *Am-h^RNAi^* embryo stained for *Am-eve* RNA showing loss and disruption of central stripes, but an anterior stripe and posterior stripes of cells expression of *Am-eve* still remain. (I) *Am-h^RNAi^* knockdown embryo stained for *Am-run* RNA showing over-expression of *Am-run* RNA throughout the embryo except in the anterior terminus. Fluctuations in the intensity of staining indicate some segmental modulation of RNA expression. (J). *Am-ftz* ^RNAi^ embryo stained for *Am-eve* RNA showing disruption of anterior stripes of *Am-eve* leading to fusion of the first 4 stripes of *Am-eve* expression and three posterior stripes of cells expressing *Am-eve*. (K) *Am-run* stained *Am-ftz* ^RNAi^ embryo showing loss of anterior stripes of expression, and disorganized posterior ones. (L) *Am-h* stained *Am-ftz* ^RNAi^ embryo with disorganized stripes and increased background staining.

In *Tribolium*, RNAi knockdown of either *Tc-eve* or *Tc-run* produces larvae lacking abdominal segments, due to a pair-rule regulatory circuit in which pair-rule genes activate each other's expression [16]. Since knockdown of *Am-eve* and *Am-run* also produces larvae with loss of segments and segment polarity gene expression, we examined the effect of knockdown on the interplay of gene regulation between these genes (Figure 6) to determine if a similar circuit is present.

*Am-eve* (Figure 6A and [20]), *Am-run* (Figure 6B and [27]) and *Am-h* (Figure 6C) are expressed during segmentation as dual segment periodicity stripes, which form in anterior posterior sequence and then split to form segmental stripes during stage 5 and 6).

In *Am-eve* ^RNAi^ embryos, no striped expression of *Am-run* or *Am-h* (Figure 6D–E) occurs. In *Am-run* ^RNAi^ embryos, *Am-eve* expression collapses to a single abdominal stripe of cells (Figure 6F), stripes of *Am-h* are disrupted (Figure 6I). In *Am-h* ^RNAi^ embryos *Am-eve* stripes are reduced in central regions (Figure 6H), *Am-run* becomes ubiquitously expressed, with striped variation in expression levels, in anterior and central regions (Figure 6I). In *Am-ftz* ^RNAi^ embryos, *Am-eve* stripes are reduced in the anterior and fused in central regions (Figure 6J). *Am-run* stripes become poorly defined and *Am-run* RNA is present as low levels throughout the embryo (Figure 6K). Ubiquitous expression of *Am-h*, with striped variation in expression levels, occurs in *Am-ftz* ^RNAi^ embryos (Figure 6L).

These experiments indicate considerable cross-talk between pair-rule genes but also show that many of the defects observed in RNAi phenotypes of pair-rule gene orthologues are more extensive than might be expected if they were acting only as pair-rule genes. This makes it very difficult to determine if the effect of gene knock-down in each experiment is direct, through regulation of that pair-rule gene, or indirect, through regulation of some early part of the segmentation process.

### Do honeybee pair-rule gene orthologues regulate gap gene expression?

The early maternal expression of *Am-eve*, *Am-ftz* and *Am-run*, and the severe effects on both anterior/posterior and dorso/ventral patterning, imply that these genes may have significant patterning roles early in development. To test this possibility we examined the effect of RNAi knockdown of these genes on the expression of previously identified honeybee gap genes [20], [30], [32].

**Figure 7:**
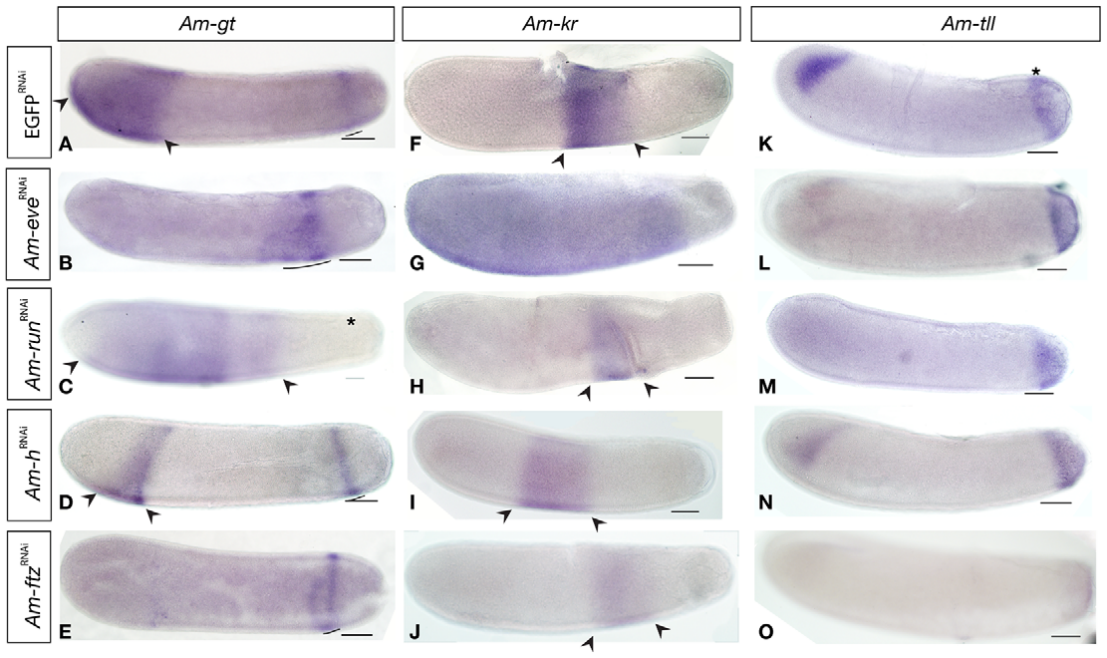
Gap gene expression in pair-rule gene knockdown embryos at stage 4. All embryos are oriented with anterior to the left and dorsal side up. Scale bars are 200 µm. (A) *Am-gt* is expressed in an anterior domain (arrowheads) and a posterior stripe (line). (B) is reduced in *Am-eve* ^RNAi^ embryo showing reduction in *Am-gt* expression into a broad abdominal domain (line). (C) *Am-run* ^RNAi^ embryos stained for *Am-gt* RNA displaying loss of the posterior stripe of *Am-gt* RNA(asterisk), and expansion of the anterior domain towards the posterior, as well as some retraction of staining from anterior and dorsal regions of the embryo (arrowheads). (D) *Am-h* ^RNAi^ embryo stained for *Am-gt* RNA displaying reduction of the anterior domain (arrowheads). (E) *Am-ftz* ^RNAi^ embryo stained for *Am-gt* RNA indicating loss of the anterior domain, and disruption of the posterior stripe. (F) *Am-kr* is expressed in a broad domain in central regions of the embryo. (G) *Am-kr* expression in an *Am-eve* ^RNAi^ embryo showing reduction in intensity of staining but expansion of the Am-kr domain to the entire embryo excepting the posterior pole. (H) The *Am-kr* expression domain in an *Am-run* ^RNAi^ embryo is shifted slightly towards the posterior (arrowheads). (I) *Am-kr* expression in an *Am-h* ^RNAi^ embryo is slightly shifted to the anterior of the normal expression domain. (J) *Am-kr* RNA stained *Am-ftz* ^RNAi^ embryo showing the Am-kr expression domain (arrowheads) shifted toward the posterior. (K) *Am-tll* RNA is expressed in an anterior triangular domain (arrow) and a posterior cap (asterisk). (L). *Am-eve* ^RNAi^ embryo stained for *Am-tll* RNA showing loss of the anterior domain of *Am-tll*. (M). *Am-tll* RNA expression in an *Am-run* ^RNAi^ embryos showing loss of the anterior domain of expression. (N) *Am-h* ^RNAi^ embryostained for *Am-tll* RNA showing no difference from control embryos. (O) *Am-ftz* ^RNAi^ embryo stained for *Am-tll* RNA showing no anterior expression domain and weak staining in the posterior cap.

*Am-gt* is required for patterning the anterior of honeybee embryos [20]. At stage 4, *Am-gt* RNA is detected in a thoracic domain and a posterior stripe (Figure 7A and [20]). In *Am-eve* ^RNAi^ (Figure 7B) embryos, the *Am-gt* expression domain in the anterior of the embryo is absent, while the posterior domain expands towards the anterior, especially in ventral regions of the embryo. In *Am-run* ^RNAi^ (Figure 7C) embryos, the anterior domain of *Am-gt* expression does not extend as dorsally as in control embryos, but the domain extends toward the posterior, with RNA detected in abdominal regions of the embryo. The anterior domain of *Am-gt* also does not extend as far to the anterior as in control embryos. The posterior stripe of *Am-gt* is absent in *Am-run* ^RNAi^ embryos (asterisk, Figure 7C).

In *Am-h* ^RNAi^ stage 4 embryos, *Am-gt* expression is similar to wild-type, except the anterior domain forms a slightly different shape and perhaps doesn't extend as far anterior as in control embryos (Figure 7D arrowheads).

In *Am-ftz* ^RNAi^ embryos, the anterior domain of *Am-gt* RNA expression is absent, while the posterior stripe is still present, though often disrupted or slightly expanded (Figure 7E).

*Am-kr* is expressed in a central domain in stage 4 embryos where it acts to pattern thoracic and abdominal segments (Figure 7F and [20]). Knockdown of *Am-eve* leads to weak over-expression of *Am-kr* RNA throughout the embryo excepting the posterior pole (Figure 7G).

Knockdown of *Am-run* expression (Figure 7H) results in a slight reduction in the extent of the *Am-kr* expression domain.

Knockdown of *Am-h* has little affect on the expression of *Am-kr*, though in some embryos (as in that pictured), the domain is shifted slightly to the anterior (Figure, 7I).

Knockdown of *Am-ftz* appears to reduce the both intensity and the width of the expression domain of *Am-kr* (Figure 7J).

*Am-tll* is required for terminal patterning and is expressed in a posterior cap of cells and triangular anterior domain at stage 6 (Figure 7K and [30]). In *Am-eve* ^RNAi^ embryos, the anterior domain of *Am-tll* RNA expression is absent, while the posterior cap appears unaffected (Figure 7L). In some *Am-eve* ^RNAi^ specimens (data not shown), the anterior domain is fainter, but not entirely absent.

In *Am-run* ^RNAi^ embryos, the anterior domain of *Am-tll* expression is absent, and the posterior domain unaffected (Figure 7M). *Am-h* ^RNAi^ does not have any appreciable affect on *Am-tll* RNA expression (Figure 7N). In *Am-ftz* ^RNAi^ embryos, expression of *Am-tll* is reduced to faint expression in the posterior, and absent from the anterior of the embryo (Figure 7O).

RNAi knockdown of all of the pair-rule gene orthologues we have examined show some effect on the expression of gap genes in the honeybee. In the case of *Am-h*, these effects are slight. For *Am-ftz*, *Am-run* and *Am-eve*, the range and scale of the defects in gap gene expression patterns led us to speculate that these pair-rule gene orthologues may be acting to modify the expression of maternal coordinate genes.

### Do honeybee pair-rule gene orthologues regulate maternal coordinate gene expression?

To test this possibility we examined the expression of three previously identified maternal coordinate genes in pair-rule gene knockdown embryos.

**Figure 8:**
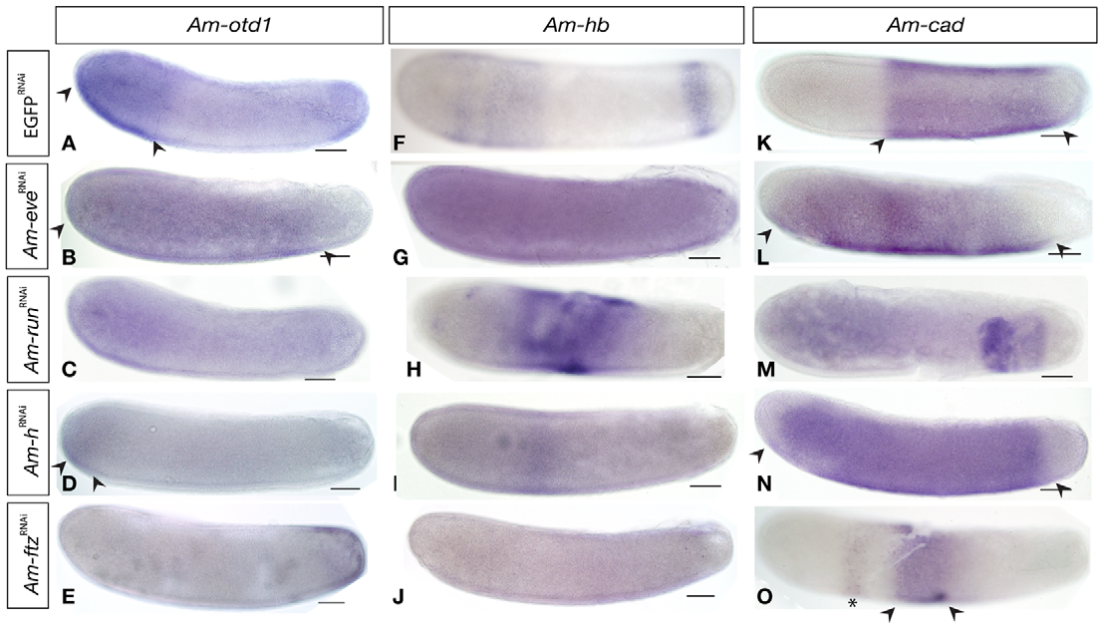
Expression of maternal patterning genes in pair-rule orthologue RNAi embryos at stage 4. All embryos are oriented with anterior left and dorsal up. Scale bars are 200 µm. (A) Expression of *Am-otd1* RNA in stage 4 *EGFP* ^RNAi^ embryos is in a weak anterior domain and posterior cap. (B). In an *Am-eve* ^RNAi^ embryo, *Am-otd1* RNA is expressed in the anterior three-quarters of the embryo (arrowheads), but is absent from the posterior. (C). In an *Am-run* ^RNAi^ embryo, *Am-otd1* RNA is expressed throughout the embryo. (D) *Am-h* ^RNAi^ embryo stained for *Am-otd1* RNA showing reduction in expression of both anterior and posterior expression domains. (E) *Am-ftz* ^RNAi^ embryo stained for *Am-otd1* RNA showing loss of the anterior domain of expression while posterior domain is retained (asterisk). (F) *Am-hb* is expressed in an anterior domain in thoracic regions and a posterior stripe (arrow). (G) *Am-hb* RNA expression in a *Am-eve* ^RNAi^ embryo, showing overexpression of *Am-hb* throughout the embryo. (H) *Am-hb* RNA is expressed at higher levels in a disorganized central domain in an *Am-run* ^RNAi^ embryo. (I) *Am-hb* expression in an *Am-h* ^RNAi^ embryo, showing reduction in expression in both domains. (J) *Am-hb* RNA is absent throughout the entire embryo in an *Am-ftz* ^RNAi^ embryo. (K) *Am-cad* is expressed in a broad domain in central, abdominal regions of the honeybee embryo (arrowheads). (L) *Am-eve* ^RNAi^ embryo showing *Am-cad* RNA expression extending more towards the anterior (arrowheads) and reduced in dorsal regions than in control embryos. (M) *Am-run* ^RNAi^ embryo showing *Am-cad* expression reduced to posterior domain with the same posterior boundary as in control embryos. (N) *Am-h* ^RNAi^ embryo stained for *Am-cad* expression showing expansion into anterior regions (arrowheads) with no change in the posterior boundary. (O) *Am-cad* expression in an *Am-ftz* ^RNAi^ embryo showing expression in a central abdominal band (arrows) and weak anterior stripe (asterisk).

In stage 4 embryos the RNA from the anterior patterning gene *Am-otd1* is detectable in cells in the anterior third of the embryo (between the arrowheads in Figure 8A) and, weakly, at the posterior terminus (Figure 8A and [32]). In *Am-eve* ^RNAi^ embryos, *Am-otd1* RNA is found throughout the embryo, being absent only in a small domain at the posterior terminus (Figure 8B arrowheads). *Am-run* ^RNAi^ knockdown results in weak staining for *Am-otd1* RNA throughout the embryo (Figure 8C). *Am-otd1* staining appears unchanged in *Am-h* ^RNAi^ embryos with both anterior (arrowheads) and posterior domains showing no effect of knockdown (Figure 8D). In *Am-ftz* ^RNAi^ embryos, the anterior domain of cells expressing of *Am-otd1* mRNA is absent, but the posterior stripe of expression is present as in control embryos (Figure 8E).

*Am-hb*, a regulator of anterior and thoracic development [32], is expressed at stage 4 in a distinct thoracic stripe of cells, and in a posterior stripe. (Figure 8F and [32]). *Am-eve* ^RNAi^ embryos show overexpression of *Am-hb* expression throughout the embryo at stage 4 (Figure 8G). *Am-run* ^RNAi^ knockdown leads to variable effects on *Am-hb* expression, producing a disorganized central domain of strong *Am-hb* expression, with no posterior stripe. The extent of this central domain varies between injected embryos (Figure 8H). *Am-h* ^RNAi^ embryos show weaker than control staining of the posterior stripe of *Am-hb*, and slight effects on the thoracic domain, often narrowing it as shown in Figure 8I. *Am-ftz* ^RNAi^ embryos have no expression of *Am-hb* RNA although occasional specimens show a faint posterior stripe (Figure 8J).

*Am-cad* RNA is expressed in cells in a broad abdominal-posterior domain, not including the posterior terminus, where it acts to pattern abdominal and posterior regions (Figure 8K arrowheads and [20]). In *Am-eve* ^RNAi^ embryos *Am-cad* RNA spreads more anteriorly than in control embryos, but still with the same posterior boundary (Figure 8L, arrowheads). Expression also does not extend as far dorsally as in controls. In *Am-run* ^RNAi^ knockdown embryos, *Am-cad* RNA is present in a much smaller posterior domain than control embryos, not extending as far anterior, or dorsally, but with the same posterior boundary. This smaller expression domain often has different intensities of staining for *Am-cad* RNA (Figure 8M). In *Am-h* ^RNAi^ embryos, *Am-cad* RNA extends further into the anterior than in control embryos, while still respecting the posterior boundary (Figure 8N, arrowheads). In *Am-ftz* ^RNAi^ embryos, *Am-cad* expression is reduced to cells in a broad band in the central regions of the embryo of the embryo (Figure 8O arrowheads), with a faint stripe of cells expressing *Am-cad* RNA anterior to it (Figure 8O asterisk).

The implication of the phenotypes of pair-rule orthologue gene knockdown on maternal coordinate gene expression is that these genes are having unexpected early patterning roles in honeybee embryos.

## Discussion

### Pair-rule gene orthologues and maternal patterning

The expression patterns and RNAi knockdown phenotypes of *Am-eve*, *Am-run* and *Am-ftz* indicate these genes are acting in early patterning of the embryo, as well as in later segmentation. All are expressed maternally, with maternal RNA persisting into early embryonic stages, providing the potential for early patterning roles. Our double-stranded RNA injections in just-laid embryos are likely triggering RNAi-mediated knockdown of this maternally deposited RNA, as well as affecting later striped patterns of zygotic expression. The phenotypes we see are thus reflections of the role of the RNA in the early embryo, not the oocyte.

*Am-eve*, *Am-run* and *Am-ftz* all have early patterning roles, affecting the expression of key maternal genes, as well as gap, pair-rule and segment polarity genes. These early patterning defects make a clear interpretation of their interactions during segmentation difficult.

*Am-h*, despite having a role in limiting *Am-cad* expression and perhaps affecting hunchback expression, appears to act mainly in segmentation, as these early roles leave little phenotypic effect when knocked down.

*Am-eve* normally represses *Am-otd1* and *Am-hb*, as both are over expressed in *Am-eve* ^RNAi^ embryos. A change in the expression of these maternal patterning genes probably explains the severe *Am-eve* ^RNAi^ phenotypes. *Am-ftz* has a role in anterior patterning, probably through activation of both *Am-otd1* and *Am-hb*. *Am-run* appears to be a regulator of posterior development, repressing the posterior domains of both *Am-hb* and *Am-otd1*. It is not clear if these interactions are direct or mediated through other factors.

Maternal roles for these genes have not been described in other insects, although maternally expressed genes with a pair-rule mutant phenotype have been described in *Drosophila* [34]–[38]. In these cases, maternal expression produces a co-factor for a zygotic pair-rule gene, regulating specific pair-rule genes or acting in cuticle development.

In *Drosophila*, *run* has been shown to act with gap gene properties by antagonizing transcriptional activation by Bicoid [39]. The effects, however, are slight, but do suggest that this gene may act more generally in segmentation.

In the cricket *Gryllus* and in the milkweed bug *Oncopeltus*, *eve* orthologues have earlier roles in segmentation, producing gap gene like effects [6], [7]. This is thought to be due to the broad expression domain of *eve* that appears in central regions of these embryos and then splits into stripes. Our results are similar, but *Am-eve* in the honeybee acts earlier, regulating maternal genes as well as gap genes.

Despite these examples, the case of three pair-rule gene orthologues having major patterning roles in early development is unique. Two possible explanations exist. Either these maternal roles are ancestral ones, supported by the early roles for *eve* in *Oncopeltus* [6] and *Gryllus* [7], or these three genes have been co-opted into maternal patterning in the lineage leading to honeybees, and these roles are likely to be specific to that lineage. In *Oncopeltus* and *Gryllus*, gap gene functions of *eve* have been suggested as being due to the broad initial domain of *eve*, which then splits into stripes [6], [7]. This expression domain is also present in honeybee *Am-eve* expression [20], and is not equivalent to the maternal expression of *Am-eve*, *Am-ftz* and *Am-run* we have presented here. We hypothesize, therefore, that the maternal expression of these three pair-rule gene orthologues is due to co-option of these genes into maternal patterning in the honeybee lineage.

It is interesting to note the activity of *Am-ftz* in this regard. *Ftz* is a so-called ‘rogue’ hox gene [10], related to Hox 6, with roles in segmentation in insects. *Ftz* has changed its expression pattern, implying a change in its function, multiple times in Arthropod evolution [40]–[42]. *Am-ftz* acts in maternal patterning to regulate anterior development, probably through regulating both *Am*-*hb* and *Am-otd1*. This draws an interesting analogy with *Drosophila bicoid*, also a rogue *Hox* gene, though in this case related to Hox 3 rather than Hox 6, which has also taken up a maternal role in *Drosophila* anterior patterning and regulates both *hunchback* [43] and *otd* [44] (*ocelliless* (*oc*) in *Drosophila*). While *Am-ftz* does not have the Glutamine to Lysine substitution at position 50 of the homeodomain seen in bicoid and related to its evolution from a Hox 3 ancestor [45], it is intriguing that a Hox gene is also found in bees with a maternal, anterior patterning role.

### Roles in segmentation and pair-rule patterning

Despite the early patterning roles for these pair-rule gene orthologues in honeybees, it is clear they also function in segmentation. All are expressed with dual segment periodicity, with broad stripes appearing across two segments, which then split to form single stripes, as seen in *Gryllus* [7], and *Schistocerca* [12].

In honeybees, the maternal role for many of these genes obscures their activity in segmentation as it is not clear if the defects in pair-rule gene expression reflect a direct regulation event, or if they are the consequences of earlier patterning deficits.

### Changing roles for conserved genes in evolution

Our finding of maternal patterning roles for conserved pair-rule genes in the honeybee indicates the propensity of such genes to be co-opted to new functions and new expression domains during evolution. The cooption of these genes into an ancestral process implies that the re-use of conserved genes in novel processes may be a common process that must be taken into account if we are to understand how developmental processes evolve.

## Supporting Information

Figure S1 **Patterning of extraembryonic membranes in stage 5 pair-rule gene orthologue knockdown embryos.** All embryos are oriented with anterior left and dorsal up. Scale bars are 100 µm. (A) Expression of *Am-zen* RNA in control, EGFP^RNAi^, embryos. *Am-zen* is expressed in anterior-dorsal regions with a stripe along the dorsal surface of the embryo. (B) Embryo in (A) stained with DAPI. Extra-embryonic membranes are distinguishable from the embryo proper by less densely spaced nuclei. In *Am-eve* ^RNAi^ embryos, *Am-zen* expression is reduced to a small domain in the dorsal posterior (arrow), (C) and the extra-embryonic membranes (D) are reduced. DAPI staining also reveals cell loss from the germband anlagen at the anterior (arrows). (E) Expansion of *Am-zen* expression from its normal dorsal domain occurs in *Am-run* ^RNAi^ embryos, associated with expansion of the extra-embryonic membranes (F). (G) *Am-h* ^RNAi^ embryos have widespread expansion of *Am-zen* expression, spreading to the ventral surface at the anterior (arrow). (H) DAPI stain of the embryo in G reveals expansion of extra-embryonic membranes. (I). *Am-ftz* ^RNAi^ embryos have no *Am-zen* expression in the anterior. Weak expression is detected in the posterior dorsal regions (arrow). (J) Loss of *Am-zen* expression is associated with loss of extraembryonic membranes.
(TIF)

Table S1 Genes, Accession numbers and *Drosophilia* orthologues discussed in this study.
(DOCX)
