# Supplementary figures and images for: Pair-Rule Gene Orthologues Have Unexpected Maternal Roles in the Honeybee (Apis mellifera)
Source: PLoS One. 2012 Sep 28;7(9):e46490. doi: 10.1371/journal.pone.0046490 (PMC3460886; doi:10.1371/journal.pone.0046490)

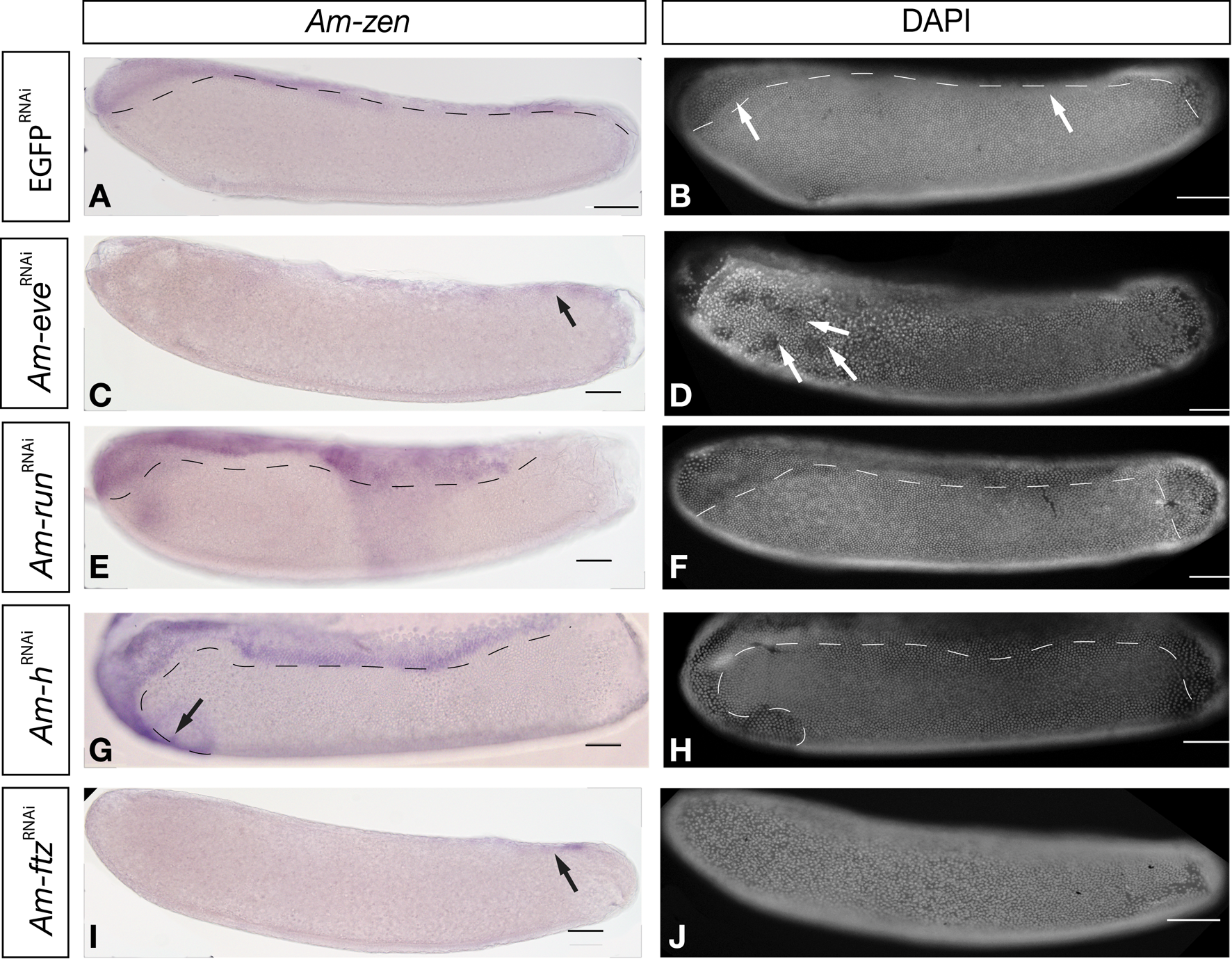

Supplement: Figure S1 — Patterning of extraembryonic membranes in stage 5 pair-rule gene orthologue knockdown embryos. All embryos are oriented with anterior left and dorsal up. Scale bars are 100 µm. (A) Expression of Am-zen RNA in control, EGFPRNAi, embryos. Am-zen is expressed in anterior-dorsal regions with a stripe along the dorsal surface of the embryo. (B) Embryo in (A) stained with DAPI. Extra-embryonic membranes are distinguishable from the embryo proper by less densely spaced nuclei. In Am-eve RNAi embryos, Am-zen expression is reduced to a small domain in the dorsal posterior (arrow), (C) and the extra-embryonic membranes (D) are reduced. DAPI staining also reveals cell loss from the germband anlagen at the anterior (arrows). (E) Expansion of Am-zen expression from its normal dorsal domain occurs in Am-run RNAi embryos, associated with expansion of the extra-embryonic membranes (F). (G) Am-h RNAi embryos have widespread expansion of Am-zen expression, spreading to the ventral surface at the anterior (arrow). (H) DAPI stain of the embryo in G reveals expansion of extra-embryonic membranes. (I). Am-ftz RNAi embryos have no Am-zen expression in the anterior. Weak expression is detected in the posterior dorsal regions (arrow). (J) Loss of Am-zen expression is associated with loss of extraembryonic membranes. (TIF) [file pone.0046490.s001.tif]
