# Supplementary material for: Pair-Rule Gene Orthologues Have Unexpected Maternal Roles in the Honeybee (Apis mellifera)
Source: PLoS One. 2012 Sep 28;7(9):e46490. doi: 10.1371/journal.pone.0046490 (PMC3460886; doi:10.1371/journal.pone.0046490)
Supplement: Table S1 — Genes, Accession numbers and Drosophilia orthologues discussed in this study. (DOCX) [file pone.0046490.s002.docx]

| **Gene Name** | **Beebase Accession** | **NCBI Accession** | **Drosophila orthologue** | **Reference** |
| --- | --- | --- | --- | --- |
| *Am-ftz* | GB18940 | Submitted | *fushi-taratzu* | [[1](#_ENREF_1)] |
| *Am-eve* | GB10623 | JN048132 | *even-skipped* | [[2](#_ENREF_2)] |
| *Am-h* | GB14857 | Submitted | *hairy* | *This paper* |
| *Am-run* | GB11654 | XM_001121886 | *runt* | [[3](#_ENREF_3)] |
| *Am-e30* | GB11694 | M29490 | *engrailed* | [[4](#_ENREF_4)] |
| *Am-zen* | GB18792 | XP_001120101 | *zerknüllt* | [[5](#_ENREF_5)] |
| *Am-gt* | GB16015 | JN048129 | *giant* | [[2](#_ENREF_2)] |
| *Am-kr* | GB16053 | NM_001252424 | *krüppel* | [[2](#_ENREF_2)] |
| *Am-tll* | GB20053 | XM_001121187 | *tailless* | [[6](#_ENREF_6)] |
| *Am-otd1* | GB16866 | JN048126 | *ocelliless* | [[7](#_ENREF_7)] |
| *Am-hb* | GB19977 | NM_001252426 | *hunchback* | [[7](#_ENREF_7)] |
| *Am-cad* | GB10821 | JN048131 | *caudal* | [[2](#_ENREF_2)] |

*References*

1. Dearden PK, Wilson MJ, Sablan L, Osborne PW, Havler M, et al. (2006) Patterns of conservation and change in honey bee developmental genes. Genome Research 16: 1376-1384.

2. Wilson MJ, Havler M, Dearden PK (2009) Giant, Kruppel, and caudal act as gap genes with extensive roles in patterning the honeybee embryo. Dev Biol.

3. Duncan EJ, Wilson MJ, Smith JM, Dearden PK (2008) Evolutionary origin and genomic organisation of runt-domain containing genes in arthropods. BMC Genomics 9: 558.

4. Walldorf U, Fleig R, Gehring WJ (1989) Comparison of homeobox-containing genes of the honeybee and Drosophila. Proc Natl Acad Sci U S A 86: 9971-9975.

5. Osborne P, Dearden PK (2005) Non-radioactive in situ hybridisation to honeybees embryos and ovaries. Apidologie 36: 113-118.

6. Wilson MJ, Dearden PK (2009) Tailless patterning functions are conserved in the honeybee even in the absence of Torso signaling. Dev Biol 335: 276-287.

7. Wilson MJ, Dearden PK (2011) Diversity in insect axis formation: two orthodenticle genes and hunchback act in anterior patterning and influence dorsoventral organization in the honeybee (Apis mellifera). Development 138: 3497-3507.
